# A comparative approach of machine learning models to predict attrition in a diabetes management program

**DOI:** 10.1371/journal.pdig.0000930

**Published:** 2025-07-07

**Authors:** Samantha Kanny, Grisha Post, Patricia Carbajales-Dale, William Cummings, Janet Evatt, Windsor Westbrook Sherrill

**Affiliations:** Clemson University, Clemson, South Carolina, United States of America; Aga Khan University - Kenya, KENYA

## Abstract

Approximately 11.6% of Americans have diabetes and South Carolina has one of the highest rates of adults with diabetes. Diabetes self-management programs have been observed to be effective in promoting weight loss and improving diabetes knowledge and self-care behaviors. The ability to keep vulnerable individuals in these programs is critical to helping the growing diabetic population. Utilizing machine learning is gaining popularity in healthcare settings. The objective of this study is to assess the effectiveness of several machine learning methods in predicting attrition from a diabetes self-management program, utilizing participant demographics and various evaluation measures. Data were collected from participants enrolled in Health Extension for Diabetes (HED). Descriptive statistics were used to examine HED participant demographics, while Mann-Whitney U tests and chi-square tests were used to examine relationships between demographics and pre-program evaluation measures. Through the various analyses, health-related measures – specifically the SF-12 quality of life scores, Distressed Communities Index (DCI) score, along with demographic factors (race, age, height, and educational attainment), and spatial variables (drive time to the nearest grocery store) emerged as influential predictors of attrition. However, the machine learning models showed poor overall performance, with AUC values ranging from 0.53 – 0.64 and F-1 scores between 0.19 – 0.36, indicating low predictive power. Among the models tested, XGBoost with downsampling yielded the highest AUC value (0.64) and a slightly higher F-1 score (0.36). To enhance model interpretability, SHAP (SHapley Additive exPlanations) was applied. While these models are not suitable for accurately predicting individual attrition risk in diabetes self-management programs, they identify potential factors influencing dropout rates. These findings underscore the difficulty for models to accurately predict health behavior outcomes, highlighting the need for future research to improve predictive modeling to better support patient engagement and retention.

## Author summary

Approximately 11.6% of Americans have diabetes, and South Carolina has one of the highest diabetes rates in adults. Diabetes self-management programs have proven effective, making it critical to retain vulnerable individuals in these programs to address the growing diabetic population. Machine learning is gaining popularity in healthcare settings due to its ability to predict patient outcomes. This study examined the effectiveness of machine learning to predict attrition from a diabetes self-management program, utilizing participant demographics and evaluation measures. Despite the inability of all models to accurately predict individual attrition risk in diabetes self-management programs, the models did identify quality of life scores, the Distressed Communities Index (DCI) score, race, age, height, and drive time to nearest grocery store as influential features in predicting attrition. In healthcare, datasets are commonly imbalanced due to the low prevalence of behavior-based outcomes such as treatment nonadherence or program attrition, which poses challenges for training machine learning models. While balancing the dataset slightly improved attrition prediction, the models still indicated a weak predictive ability. These findings underscore the difficulty for models to accurately predict health behavior outcomes, highlighting the need for future research to improve predictive modeling to better support patient engagement and retention.

## Introduction

An estimated 11.6% of Americans have diabetes, with 29.7 million people with diagnosed diabetes and 8.7 million people with undiagnosed diabetes [1]. Further, one in three Americans have prediabetes [2]. In South Carolina, an estimated 531,124 (13.2%) people have been diagnosed with diabetes and 123,000 (9%) have undiagnosed diabetes [3]. Research suggests that rates of diabetes will continue to rise, with a predicted 22.2% increase in diabetes prevalence indicated by 2045 [4,5]. Thus, effective diabetes self-management programs are critical to address the increasing rates of individuals being diagnosed with diabetes.

Diabetes self-management is thought to be the foundation to the overall management of diabetes [6]. The burden of the chronic condition falls on the individual to modify or maintain the behaviors associated with diabetes once their medical regime has been established and they have received a diabetes education [7]. Poor diabetes self-management has been associated with poor health outcomes [8]. Some of the most common barriers to diabetes self-management include lack of knowledge about diabetes, identifying diabetes-related medications and understanding diabetes-related prescriptions, poor continuity of care, and lack of awareness of target blood glucose and blood pressure [9]. The Task Force to Revise the National Standards for Diabetes Self-Management Education Programs, a group established to represent national diabetes-related organizations, has described diabetes self-management education and support (DSMES) programs as “the cornerstone of treatment for all people with diabetes” [10].

A Consensus Report of the American Diabetes Association, the Association of Diabetes Care & Education Specialists, the Academy of Nutrition and Dietetics, the American Academy of Family Physicians, the American Academy of PAs, the American Association of Nurse Practitioners, and the American Pharmacists Association states that DSMES “addresses the comprehensive blend of clinical, educational, psychosocial, and behavioral aspects of care needed for daily self-management and provides the foundation to help all people with diabetes navigate their daily self-care with confidence and improved outcomes” [11]. Previous research has shown that DSMES programs can promote weight loss, reduce mortality and health care costs, increase knowledge and healthy coping, and improve diabetes self-care behaviors, quality of life and A1C levels [12,13]. Despite the benefits seen from participating in DSMES programs, attendance in these programs remain low and high attrition rates have been observed in vulnerable populations [14]. Factors associated with attrition include socioeconomic status, education level, transportation and current work status [15–18]. Other factors confidence in individuals’ ability to manage their diabetes, low perceived serious of diabetes and lack of familiarity toward the program and its resources [14]. High rates of attrition hold significant implications toward DSMES and effect DSMES programs’ ability to help improve participants’ self-management skills.

Machine learning is gaining popularity in healthcare settings due to its ability to predict patient outcomes. Logistic regression is a longstanding regression technique favored by researchers for its practical utility and interpretability. This method has proven especially valuable in predicting outcomes within the healthcare sector. In contrast, other machine learning algorithms, though well-established across various research domains, are relatively newer to the healthcare field [19]. Among these, random forest classification stands out as an effective tool. It employs an ensemble of decision trees to predict critical health outcomes, such as diabetes incidence and behaviors, as well as factors like employee attrition [20,21]. Although random forest models do not offer the straightforward interpretability of logistic regression, their superior predictive capabilities increasingly attract healthcare researchers to adopt this approach. Another widely used machine learning technique is XGBoost, which is also an ensemble method of decision trees. Unlike random forests, XGBoost builds trees sequentially, with each new tree aimed at correcting the errors of its predecessors. XGBoost has recently emerged as a powerful technique for forecasting diabetes due to its high predictive accuracy.

Previous literature examining the effectiveness of machine learning in diabetes research has focused on its ability to predict diabetes based on various risk factors. There is a paucity in current diabetes literature applying machine learning techniques to predict attrition from diabetes management programs. The objective of this study is to compare several machine learning models in predicting attrition from a diabetes self-management program, utilizing participant demographics and various evaluation measures. This research incorporates novel data sources, including GIS (Geographic Information Systems) derived variables such as driving time to the nearest pharmacy, grocery store or hospital. Although these spatial barriers have been shown to negatively affect glycemic control, they are rarely included in predictive models of program engagement [22]. By integrating these GIS-based measures with socioeconomic and health-related factors, and employing multiple machine learning algorithms, this study aims to offer comprehensive insights that can aid program managers and decision-makers in enhancing the implementation and effectiveness of diabetes management programs.

## Materials and methods

### Health Extension for Diabetes (HED)

Health Extension for Diabetes is a community-based DSMS program. DSMS “refers to the support that is required for implementing and sustaining coping skills and behaviors needed to self-manage on an ongoing basis” [23]. HED is a 4-month program that is comprised of eight bi-weekly core group educational sessions and personalized follow-up support sessions between educational sessions. The curriculum is developed based on the Association of Diabetes Cares and Education Specialists’ (ADCES) Seven Self-Care Behaviors for Managing Diabetes, which include healthy coping, healthy eating, being active, taking medication, monitoring, problem-solving and reducing risks [24]. The American Diabetes Association has recognized HED as a Practice-Tested Support Program [25].

HED was created through a collaboration between Clemson University, a state land-grant university, and a regional healthcare system. HED is delivered via health extension agents (HEAs) with at least one session delivered by a Certified Diabetes Care and Education Specialist (CDCES) with a health system affiliation. The health extension agents who lead the intervention have been trained in ADCES Prevention 101 and ADCES7 Diabetes Self-Care Behaviors.

### Data collection & study population

Participant demographics are collected at registration. Additionally, participants are asked a variety of social determinants of health questions including questions related to socioeconomic status, neighborhood and physical environment, food environment and accessibility, health care and social environment. Program measures are asked pre-program and post-program. These measures include nutrition behaviors, physical activity behaviors, Summary of Diabetes Self-Care Activities (SDSCA), quality of life (SF-12), self-efficacy for diabetes and diabetes knowledge. Eligibility for HED includes being 18 years of age or older, a resident of South Carolina, and diagnosed with Type 1 or Type 2 diabetes. This study received approval by Prisma Health’s IRB Review Board (Pro00073892). Formal consent was obtained verbally for all participants included in this study.

Additional variables were integrated for each participant through GIS analysis to capture the potential logistical challenges in diabetes management. Literature suggests that driving times to essential services such as grocery stores, pharmacies, and hospitals may significantly impact diabetes management [26]. Utilizing ArcGIS Pro, Version 3.3 [27] network analysis, the driving times from each participant’s address to the nearest grocery store, pharmacy, and hospital were calculated. Data on grocery stores and pharmacies were sourced from Esri Business Analyst—Business Location Data as of July 2023 [28]. Grocery stores were identified using North American Industry Classification System (NAICS) codes 44511003 (grocery-retail) and 44511009 (grocery-pickup), excluding convenience stores, yielding a total of 943 grocery store locations in South Carolina. Pharmacies were identified using NAICS code 45611009, with a total of 1,155 locations extracted. Hospital locations, totaling 108, were provided by the South Carolina Department of Health and Environmental Control as of November 2020 [29].

To further enrich the dataset, additional socioeconomic variables were incorporated using spatial overlay techniques. For instance, the Social Vulnerability Index (SVI) [30] values were assigned to each participant by spatially joining their addresses with corresponding Census Tracts. Similarly, the Distressed Communities Index (DCI) [31] values were assigned based on the participant’s zip code, enhancing our understanding of the socioeconomic factors influencing program attrition.

### Statistical analysis

Descriptive statistics for baseline participant demographics and evaluation measures were calculated and presented for continuous variables as mean (standard deviation) and for categorical variables as the number (percentage). A target variable in this analysis is binary, representing participants’ completion status in HED: those who graduated (coded as 0) and those who dropped out (attrition, coded as 1). Mann-Whitney U tests and chi-square tests were used to calculate the differences in participant demographics and evaluation measures between the target variable (participants’ completion status). Analysis was completed using SAS software, R, and Python [32–34].

### Logistic regression

Logistic regressions were used to examine whether participant demographics or evaluation measures can predict HED program attrition. Analysis was completed using Python. Logistic regression estimates coefficients for each independent variable by maximizing the likelihood of the observed outcomes, specifically, whether a participant left the program (attrition) or not. These likelihoods are then transformed using the logistic function to produce probabilities between 0 and 1.

**Fig 1:**
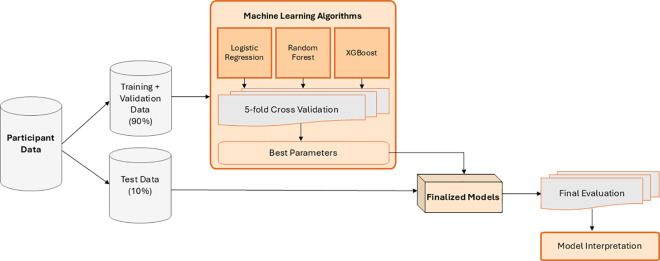
Logistic regression, random forest, and XGBoost workflow.

The dataset was divided into 90% of the data being used to train the logistic regression and 10% of the data used to evaluate the model. A grid search with five-fold cross-validation was used for all machine learning models in this study to find the optimal hyperparameters that would produce the highest area under the receiver operating characteristic curve (AUC) score during the cross-validation process. AUC was chosen as the primary metric to tune the machine learning models because it considers both true positive and false positive rates and is independent of the threshold. When predicting on the test data, a threshold was applied to classify the probabilities into binary predictions. The threshold chosen was calculated by using Youden’s J-statistic [35] to find the threshold that gives the best trade-off between true positives and true negatives. This can be calculated as sensitivity plus specificity minus one. The optimal threshold for each model was calculated by using the out-of-fold predictions in the five-fold cross validation to calculate the threshold that maximizes Youden’s J-statistic. This method of threshold tuning allows an unbiased calculation of the threshold for each model as the threshold was not calculated on the unseen test data. This train and test data split and threshold calculation was subsequently applied to all machine learning models as depicted in Fig 1.

To fine-tune the logistic regression, a five-fold cross-validation was implemented to determine the optimal hyperparameter C, a tuning parameter that controls the balance between minimizing training error and preventing model overfitting. The optimal value of C strikes a balance between simpler models, which are less prone to overfitting (*low C*), and models that fit the training data more closely (*high C*). The cross-validation process involves dividing the dataset into five subsets, training the model on four subsets, and validating it on the fifth. This process is repeated five times, with each subset used once as the validation set, ensuring a robust evaluation of model performance and the identification of the best C value. The best C value found was 0.59, which indicates moderate regularization.

### Random forest classification

As depicted in Fig 1, all models utilized the same participant data to predict attrition. In the case of random forest, the algorithm employs a subset of the participant data - the training dataset - to build an ensemble of decision trees. The remaining data serves as the test dataset, allowing evaluation of the model’s performance on unseen data.

During the model’s training phase, each decision tree is allocated a random subset of explanatory variables. Each node within these trees corresponds to a decision point based on one selected variable. As each participant from the training dataset is evaluated by a tree, the tree makes a prediction of whether that participant will drop out of the program. Importantly, each tree in the ensemble operates independently with its own subset of variables. The final model prediction represents the consensus – determined by a majority vote – among all trees in the ensemble [31].

In this study, 90% of the participant data was utilized to train the random forest model.. A random subsample of the training data was selected to train each tree. The remaining 10% of data was held out as an external test set for final model evaluation. The model was further optimized by adjusting the random forest hyperparameters: number of trees, maximum tree depth, maximum features considered when splitting each node, the minimum samples required to be in a leaf node, and minimum samples required to be split in a node. Hyperparameters were found using a grid search of set parameters to find the combination of parameters with the highest AUC score using five-fold cross-validation. Different sets of hyperparameters were tested until the average AUC from cross-validation showed no significant improvement. The optimal hyperparameters for the Random Forest model were a maximum depth of 4, a maximum of 5 features considered at each split, a minimum samples per leaf of 1, a minimum samples per split of 2, and a number of trees of 2000.

### XGBoost: eXtreme gradient boosting

XGBoost is a highly efficient and scalable machine learning algorithm that constructs an ensemble of decision trees in a sequential manner. Each tree is trained to correct the errors of the preceding trees by minimizing the residuals, thereby improving the overall predictive performance. The final prediction is obtained by aggregating the outputs of all individual trees.

To optimize the XGBoost model, hyperparameters were tuned to maximize the AUC from a grid search five-fold cross-validation before evaluating the model on the test set. The hyperparameters considered included the maximum depth of trees, learning rate, subsample, L1 and L2 regularization term on weights (alpha, and lambda), the minimum sum of instance weight needed in a decision tree built to correct errors made by previous trees, and sampling ratio of columns when making each tree. The optimal hyperparameters for the XGBoost model were a maximum depth of 1, a column subsampling ratio of 0.9, a learning rate of 0.0025, a minimum child weight of 6, and 1000 estimators. These hyperparameters result in a highly regularized model characterized by shallow tree depth, a low learning rate, and a high minimum child weight, likely selected to reduce the risk of overfitting.

### Balancing techniques: Cost-sensitive. SMOTE, and down sampling

To mitigate the imbalance of the study data, three balancing techniques were implemented: SMOTE (Synthetic Minority Over-sampling Technique), a cost-sensitive approach, and down sampling. SMOTE enhances the representation of the minority class (attrition) by generating synthetic samples, thereby creating more balanced models and improving the predictive accuracy regarding attrition [36]. The cost-sensitive approach, on the other hand, assigns a higher penalty to incorrect predictions of attrition, thereby reducing the overall cost of misclassification [37]. This method involves calculating weights for the cost-sensitive random forest by taking the inverse of the attrition ratio within the dataset. Down sampling, also known as under sampling, involves randomly reducing the size of the majority class to match that of the minority class in a dataset [37]. By reducing the majority class, this technique helps to lessen bias, which typically occurs when a model maximizes accuracy by predominantly predicting the majority class. In cross validation, all balancing techniques were applied only to the training portion of each fold to reduce the potential for data leakage.

The hyperparameters for the most optimal cost-sensitive random forest were max tree depth of 20, max features of 1, minimum samples leaf of 1, minimum samples split of 3, and a number of trees being 1000. The relatively high number of trees with low features per split may indicate that the model focuses on more individual patterns in the data. The hyperparameters for the most optimal SMOTE random forest were max tree depth of 10, max features of 5, minimum samples leaf of 1, minimum samples split of 3, and a number of trees being 500, indicating modest regularization. In comparison, the hyperparameters for the most optimal down sampled random forest were max tree depth of 5, max features of 5, minimum samples leaf of 1, minimum samples split of 2, and a number of trees being 100, which are relatively shallow and conservative. The optimal hyperparameters for the cost-sensitive XGBoost model included a learning rate of 0.005, a maximum tree depth of 1, a minimum child weight of 12, a tree number of 1,000, full column and row sampling, and regularization parameters of alpha being 0.005 and lambda being 1. The optimal hyperparameters for the SMOTE-based XGBoost model included a learning rate of 0.01, maximum tree depth of 1, number of trees being 500, full column and row sampling, and strong regularization (alpha = 1 and lambda = 2). Both the cost sensitive and SMOTE random forest are highly regularized. Lastly, the optimal hyperparameters found for the down sampled XGBoost model were a learning rate of 0.01, a maximum tree depth of 9, a number of trees of 100, 80% feature sub-sampling, 60% of data being sub-sampled, and default regularization of alpha being 0 and lambda being 1. The lower sub-sampling and feature sub-sampling indicate optimization to avoid overfitting.

### Model evaluation

Predictive machine learning performance is typically assessed using several key metrics: accuracy, sensitivity, specificity, precision, the F-1 score, and area under the curve (AUC). These metrics are derived from confusion matrices, which tabulate the discrepancies between predicted and actual attrition statuses for participants across both the training and validation datasets. **Accuracy** represents the proportion of all participants whose attrition status is correctly predicted. **Sensitivity**, or the true positive rate, indicates the percentage of participants who did not complete the program and were accurately predicted as such. **Specificity**, or the true negative rate, reflects the percentage of participants who completed the program and were correctly identified. **Precision** is the proportion of predicted positive cases that were true positives. The **F-1 score** combines precision and recall, providing a balanced measure of a model’s performance with and F-1 score of 1 indicating a perfect model prediction. Lastly, **Area Under the Curve** (AUC) of the Receiver Operating Characteristic (ROC) curve represents the probability that the model ranks a randomly selected true positive instance higher than a randomly selected true negative instance. The ROC curve plots the True Positive Rate (sensitivity) against the False Positive Rate (1 - specificity) across various threshold settings. An AUC of approximately 0.5 suggests that the model’s performance is equivalent to random guessing. AUC values between 0.5 and 0.6 indicate poor model performance, potentially due to factors such as inadequate data, class imbalance, or high noise levels [31].

### Explainable AI: Shapley Additive exPlanations (SHAP) analysis

Explainable Artificial Intelligence (EXAI) refers to a set of methodologies and design principles that aim to enhance the interpretability of machine learning models. In public health, XAI has been applied to a range of clinical decision-support tools, including disease classification, image segmentation, and the mitigation of sensor-related bias [38]. Beyond healthcare, XAI has demonstrated value in domains such as market analysis, where it has contributed to improved customer retention [39].

Among the most widely adopted XAI techniques is Shapley Additive Explanations (SHAP), a post hoc, model-agnostic method that provides insight into how individual features contribute to specific model predictions. SHAP can be applied to a variety of black-box models, including random forest and XGBoost, to trace and visualize the model’s decision-making process. Its popularity stems from its ability to offer both local (individual prediction-level) and global (overall feature importance) explanations. SHAP is grounded in cooperative game theory, using Shapley values to fairly attribute a model’s output to its input features, making it a powerful tool for understanding and communicating machine learning results in complex domains [40].

To further interpret the predictions of the best-performing model in this study, SHAP analysis was conducted to examine feature importance. SHAP values and visualizations were generated using the SHAP Python package [41].

## Results

### Descriptive statistics for participant demographics

Results for descriptive statistics for participant demographic characteristics can be found in Table 1. The average age of participants enrolled in HED was 64.16 (± 12.70). A majority of participants (N = 689; 71.55%) enrolled in HED were females and most (N = 570; 58.76%) participants were white. Some participants enrolled in HED were Hispanic/Latino (N = 71; 7.32%). Half of the participants enrolled in HED (N = 510; 52.90%) were married. Of those enrolled in HED, most were either currently working (N = 400; 41.41%) or retired (N = 379; 39.23%). Online programming was the most common type of program delivery method (N = 572; 59.21%). Many participants who enrolled in HED had a family history of diabetes (N = 749; 77.62%) or a history of hypertension (N = 709; 73.47%).

**Table 1:** Descriptive statistics for participant demographic characteristics.

Pre-Program Factors	Overall *N = 970*	HED Graduates *N = 809*	HED Attrition *N = 161*	P-Value
Age (years), Mean (Std.)	64.16 (± 12.70)	64.29 (± 12.65)	63.49 (± 12.07)	0.4652
Height (inches), Mean (Std.)	65.88 (± 4.13)	65.86 (± 4.21)	66.00 (± 3.73)	0.6891
Weight (lbs.), Mean (Std.)	211.45 (± 52.64)	211.86 (± 52.83)	209.40 (± 51.76)	0.5899
BMI $\left(kg/m^{2}\right)$, Mean (Std.)	34.29 (± 8.07)	34.30 (± 8.08)	34.28 (± 8.03)	0.9804
Biological Sex, N (%)				0.7699
Females	689 (71.55)	573 (71.36)	116 (72.50)	
Males	274 (28.45)	230 (28.64)	44 (27.50)	
Race, N (%)				0.0090*
African American or Black	318 (32.78)	281 (34.73)*	37 (22.98)	
White	570 (58.76)	462 (57.11)	108 (67.08)*	
Other	72 (7.42)	56 (6.92)	16 (9.94)	
Prefer Not to Answer	10 (1.03)	10 (1.24)	0 (0.00)	
Ethnicity				0.6617
Hispanic/Latino	71 (7.32)	57 (7.05)	14 (8.70)	
Not Hispanic/Latino	873 (90.00)	731 (90.36)	142 (88.20)	
Other	11 (1.13)	8 (0.99)	8 (1.86)	
Prefer Not to Answer	15 (1.55)	13 (1.61)	2 (1.24)	
Education, N (%)				0.0866
Less than High School	37 (3.81)	29 (3.58)	8 (4.97)	
Some High School	44 (4.54)	37 (4.57)	7 (4.35)	
High School Diploma/GED	195 (20.10)	153 (18.91)	42 (26.09)	
Some College	191 (19.69)	162 (20.02)	29 (18.01)	
Technical/Associate Degree	134 (13.81)	111 (13.72)	23 (14.29)	
Bachelor’s Degree	177 (18.25)	160 (19.78)	17 (10.56)	
Some Postgraduate Education	192 (19.79)	157 (19.41)	35 (21.74)	
Marital Status, N (%)				0.2409
Single	197 (20.44)	168 (20.90)	29 (18.13)	
Married	510 (52.90)	424 (52.74)	86 (53.75)	
Separated	24 (2.49)	19 (2.36)	5 (3.13)	
Divorced	117 (12.14)	103 (12.81)	14 (8.75)	
Widowed	116 (12.03)	90 (11.19)	26 (16.25)	
Employment Status, N (%)				0.3522
Unable to Work	94 (9.73)	73 (9.07)	21 (13.04)	
Not Working	93 (9.63)	80 (9.94)	13 (8.07)	
Working	400 (41.41)	331 (41.12)	69 (42.86)	
Retired	379 (39.23)	321 (39.88)	58 (36.02)	
Income, N (%)				0.9080
Less than $15,000	119 (12.27)	101 (12.48)	18 (11.18)	
$15,000 to $25,000	123 (12.68)	99 (12.24)	24 (14.91)	
$25,000 to $49,999	181 (18.66)	152 (18.79)	29 (18.01)	
$50,000 to $75,000	107 (11.03)	93 (11.50)	14 (8.70)	
$75,000 to $99,999	61 (6.29)	51 (6.30)	10 (6.21)	
Greater than $100,000	63 (6.49)	52 (6.43)	11 (6.83)	
Prefer Not to Answer	316 (32.58)	261 (32.26)	55 (34.16)	
Program Delivery Method, N (%)				0.7352
Online	572 (59.21)	480 (59.63)	92 (57.14)	
In-Person	328 (33.95)	272 (33.79)	56 (34.78)	
Hybrid	66 (6.83)	53 (6.58)	13 (8.07)	
Family History of Diabetes, N (%)				0.9691
No	216 (22.38)	180 (22.36)	36 (22.50)	
Yes	749 (77.62)	625 (77.64)	124 (77.50)	
History of Hypertension, N (%)				0.7606
No	256 (26.53)	212 (26.34)	44 (27.50)	
Yes	709 (73.47)	593 (73.66)	116 (72.50)	

### Pre-program results using HED evaluation measures

Participant pre-program results can be found in Table 2. The average physical component score for all participants indicates that their physical health is causing moderate disability for their overall quality of life (43.03 ± 10.96). The average mental component score for all participants indicates that their mental health is causing mild disability to their overall quality of life (50.77 ± 10.96). Diabetes self-efficacy (7.16 ± 1.75) and diabetes knowledge (76.72 ± 14.73) were observed to be good pre-program. Participants enrolled in the program ate a healthy diet an average of around 4 days per week, exercised an average of around 2 days per week, measured their blood glucose an average of around 4 days and conducted proper foot care an average of around 3 days. There were no significant differences observed for all pre-program evaluation HED measures between those who graduated from HED and those who dropped out of HED (*Ps* > 0.05).

**Table 2:** Pre-program participant results using HED evaluation measures.

Pre-Program Factors	Overall *N = 970*	HED Graduates *N = 809*	HED Attrition *N = 161*	P-Value
SF-12, Mean (Std.) *(Score Range: 0 – 100)*				
Physical Component Score (PCS)	43.03 (± 10.96)	43.03 (± 10.91)	43.04 (± 11.24)	0.9906
Mental Component Score (MCS)	50.77 (± 10.96)	50.60 (± 10.94)	51.62 (± 11.09)	0.2856
Diabetes Self-Efficacy, Mean (Std.) *(Score Range 0 – 10)*	7.16 (± 1.75)	7.19 (± 1.73)	7.05 (± 1.85)	0.3776
Summary of Diabetes Self-Care Activities, Mean (Std.) *(Score Out of 7 Days Per Week)*				
General Diet	4.06 (± 2.31)	4.06 (± 2.27)	4.05 (± 2.49)	0.9511
Specific Diet	3.83 (± 1.83)	3.81 (± 1.80)	3.91 (± 1.97)	0.5377
Exercise	2.45 (± 2.19)	2.50 (± 2.20)	2.20 (± 2.14)	0.1186
Blood Glucose Testing	4.10 (± 2.84)	4.12 (± 2.83)	3.98 (± 2.90)	0.5878
Foot Care	3.37 (± 2.39)	3.39 (± 2.39)	3.28 (± 2.36)	0.8432
Diabetes Knowledge, Mean (Std.), Mean (Std.) *(Score Range: 0 – 100)*	76.72 (± 14.73)	76.73 (± 14.73)	76.67 (± 14.74)	0.9670

### Logistic regression

Results for the logistics regressions can be found in Table 3 and Table 4. A significant difference was found between race and attrition. White participants were 1.775 times more at risk of dropping out of HED compared to African American participants (*p* = 0.0051). A significant difference was also found between education and attrition. Individuals with less than a high school education were 2.796 times more at risk of dropping out of HED compared to those with a bachelor’s degree (*p* = 0.0415). Individuals with a high school diploma or GED were 2.755 times more at risk of dropping out of HED compared to those with a bachelor’s degree (*p* = 0.0012). Individuals with a technical or associate’s degree were 2.060 times more at risk of dropping out of HED compared to those with a bachelor’s degree (*p* = 0.0362). Individuals with at least some postgraduate education were 2.156 times more at risk of dropping out of HED compared to those with a bachelor’s degree (*p* = 0.0157). No other significant differences were observed between baseline participant demographics/evaluation measures and HED program attrition (*Ps* > 0.05).

**Table 3:** Odds ratio for participant demographics characteristics (Odds Ratio Estimate, P-Value & 95% Confidence Interval).

	CRUDE MODEL				ADJUSTED MODEL			
**Pre-Program Factors**	**OR**	**P-Value**	**95% CI for OR**		**OR**	**P-Value**	**95% CI for OR**	
			**Lower**	**Upper**			**Lower**	**Upper**
Age (years)	0.995	0.4648	0.982	1.008	0.994	0.3803	0.981	1.007
Height (inches)	1.008	0.6888	0.968	1.051	1.008	0.7019	0.967	1.051
Weight (lbs.)	0.999	0.5895	0.996	1.002	0.999	0.6480	0.996	1.003
BMI $\left(kg/m^{2}\right)$	1.000	0.9804	0.979	1.021	1.000	0.9720	0.979	1.022
Biological Sex								
Females (ref)	1.00				1.00			
Males	0.945	0.7699	0.647	1.381	0.957	0.8207	0.653	1.401
Race								
African American or Black (ref)	1.00							
White	1.775	0.0051*	1.188	2.653				
Other	2.170	0.0200*	1.130	4.168				
Prefer Not to Answer	< 0.001	0.9812	< 0.001	> 999,999				
Ethnicity								
Hispanic/Latino (ref)	1.00				1.00			
Not Hispanic/Latino	0.791	0.4522	0.429	1.458	1.066	0.8758	0.477	2.386
Other	1.527	0.5673	0.358	6.509	1.894	0.4071	0.418	8.576
Prefer Not to Answer	0.626	0.5665	0.127	3.101	1.236	0.8098	0.220	6.926
Education								
Less than High School	2.596	0.0441*	1.026	6.572	2.796	0.0415*	1.040	7.513
Some High School	1.781	0.2340	0.689	4.604	2.049	0.1516	0.769	5.464
High School Diploma/GED	2.584	0.0021*	1.410	4.733	2.755	0.0012*	1.494	5.081
Some College	1.685	0.1087	0.891	3.187	1.734	0.0919	0.914	3.290
Technical/Associate Degree	1.950	0.0514	0.996	3.819	2.060	0.0362*	1.048	4.052
Bachelor’s Degree (ref)	1.00				1.00			
Some Postgraduate Education	2.098	0.0191*	1.129	3.900	2.156	0.0157*	1.156	4.020
Marital Status								
Single (ref)	1.00				1.00			
Married	1.175	0.4893	0.744	1.856	1.205	0.4269	0.761	1.910
Separated	1.525	0.4360	0.528	4.405	1.448	0.4959	0.499	4.206
Divorced	0.787	0.4930	0.398	1.560	0.801	0.5275	0.403	1.593
Widowed	1.674	0.0861	0.930	3.013	1.650	0.0969	0.914	2.981
Employment Status								
Unable to Work (ref)	1.00				1.00			
Not Working	0.565	0.1413	0.264	1.209	0.551	0.1272	0.256	1.185
Working	0.725	0.2513	0.418	1.256	0.725	0.2552	0.416	1.262
Retired	0.628	0.1037	0.359	1.100	0.623	0.1002	0.354	1.095
Income								
Less than $15,000 (ref)	1.00				1.00			
$15,000 to $25,000	1.360	0.3688	0.695	2.661	1.266	0.4955	0.642	2.498
$25,000 to $49,999	1.071	0.8346	0.565	2.030	0.924	0.8123	0.482	1.772
$50,000 to $75,000	0.845	0.6609	0.398	1.794	0.747	0.4537	0.348	1.604
$75,000 to $99,999	1.100	0.8243	0.473	2.557	0.946	0.8990	0.400	2.237
Greater than $100,000	1.187	0.6825	0.522	2.699	0.922	0.8502	0.396	2.144
Prefer Not to Answer	1.182	0.5710	0.662	2.111	1.072	0.8173	0.595	1.930
Program Delivery Method, N (%)								
Online (ref)	1.00				1.00			
In-Person	0.931	0.7000	0.647	1.340	0.983	0.9258	0.679	1.422
Hybrid	1.191	0.6091	0.609	2.331	1.242	0.5303	0.632	2.441
Family History of Diabetes								
No (ref)	1.00				1.00			
Yes	0.992	0.9691	0.661	1.489	1.010	0.9610	0.671	1.521
History of Hypertension								
No (ref)	1.00				1.00			
Yes	0.943	0.7606	0.644	1.379	0.957	0.8207	0.652	1.404

**Table 4:** Odds ratio for evaluation measures (Odds Ratio Estimate, P-Value & 95% Confidence Interval).

	CRUDE MODEL				ADJUSTED MODEL			
**Pre-Program Factors**	**OR**	**P-Value**	**95% CI for OR**		**OR**	**P-Value**	**95% CI for OR**	
			**Lower**	**Upper**			**Lower**	**Upper**
SF-12 *(Score Range: 0 – 100)*								
Physical Component Score (PCS)	1.000	0.9906	0.985	1.016	1.002	0.8414	0.986	1.017
Mental Component Score (MCS)	1.009	0.2857	0.993	1.025	1.008	0.3117	0.992	1.025
Diabetes Self-Efficacy *(Score Range 0 – 10)*	0.958	0.3773	0.870	1.054	0.951	0.3125	0.862	1.049
Summary of Diabetes Self-Care Activities *(Score Out of 7 Days Per Week)*								
General Diet	0.998	0.9510	0.927	1.074	0.999	0.9748	0.927	1.076
Specific Diet	1.030	0.5373	0.938	1.131	1.034	0.4851	0.941	1.137
Exercise	0.938	0.1191	0.865	1.017	0.937	0.1153	0.864	1.016
Blood Glucose Testing	0.983	0.5874	0.925	1.045	0.982	0.5608	0.923	1.044
Foot Care	0.980	0.5741	0.912	1.053	0.973	0.4515	0.905	1.045
Diabetes Knowledge *(Score Range 0 – 100)*	1.000	0.9669	0.988	1.011	1.001	0.8226	0.990	1.013

### Predictive capabilities of standard and balanced models with random prediction

To evaluate and compare the overall performance of the prediction models – Logistic Regression Random Forest, XGBoost, and their balanced counterparts – several key metrics were employed: Accuracy, Sensitivity (True Positive Rate), Specificity (False Negative Rate), Precision, the F-1 score, and AUC. AUC was the most prioritized metric, as it more effectively evaluates the models’ ability to distinguish between the majority and minority classes in this imbalanced dataset. Table 5 describes the metrics comparison for the models.

**Table 5:** Comparison of random prediction simulation, logistic regression, random forest, xgboost, balanced random forest, and balanced XGBoost models.

Metrics	Random Prediction Simulation	Logistic Regression	Random Forest	Random Forest Cost-Sensitive	Random Forest SMOTE	Random Forest Down Sampled	XGBoost	XGBoost Cost- Sensitive	XGBoost SMOTE	XGBoost Down Sampled
Accuracy	0.47	0.55	0.39	0.61	0.61	0.31	0.52	0.52	0.64	0.54
Sensitivity	0.47	0.53	0.67	0.27	0.40	0.73	0.47	0.47	0.47	0.73
Specificity	0.47	0.56	0.33	0.68	0.65	0.39	0.53	0.53	0.68	0.50
Precision	0.16	0.20	0.17	0.15	0.19	0.20	0.17	0.17	0.23	0.23
F-1 Score	0.23	0.29	0.27	0.19	0.26	0.31	0.25	0.25	0.31	0.36
AUC	0.50	0.57	0.53	0.54	0.54	0.56	0.56	0.54	0.60	0.64

A random prediction simulation was first modeled to serve as a baseline to compare against, which had an expected AUC of 0.50. Logistic regression performed better than random prediction with a higher AUC score (0.57) and F-1 score (0.23), indicating improved interpretability of attrition. The base random forest model demonstrated similar performance compared to the SMOTE random forest, cost-sensitive random forest and down sampled random, with AUC scores of.0.53 to 0.56 and F-1 scores ranging from 0.19 to 0.31. The standard XGBoost model and cost sensitive XGBoost model performed similarly to the logistic regression and random forest models with no marginal improvement to predicting attrition, while the SMOTE balanced XGBoost had a slightly higher AUC score of 0.60 and F-1 score of 0.31. The down sampled XGBoost model was found to have the highest AUC score, and F-1 score when performed on the test data with an AUC score of 0.64 and an F-1 score of 0.36. The down sampled XGBoost may have performed better due to potentially being less prone to overfitting on the test data. The overall accuracy of the models ranged from 0.31 to 0.64. This wide variation of accuracy can be attributed to the use of Youden’s J-statistic to determine the optimal classification threshold from the cross validation, prioritizing the balance between specificity and sensitivity. Thus, accuracy should not be interpreted as an overall indicator of model performance in this study. The relatively low AUC (ranging from 0.53 to 0.64) and F-1 scores (ranging from 0.19 to 0.36) suggest that the models exhibit poor discrimination in predicting attrition [42]. This indicates that the models are not effectively differentiating between the positive and negative classes.

**Fig 2:**
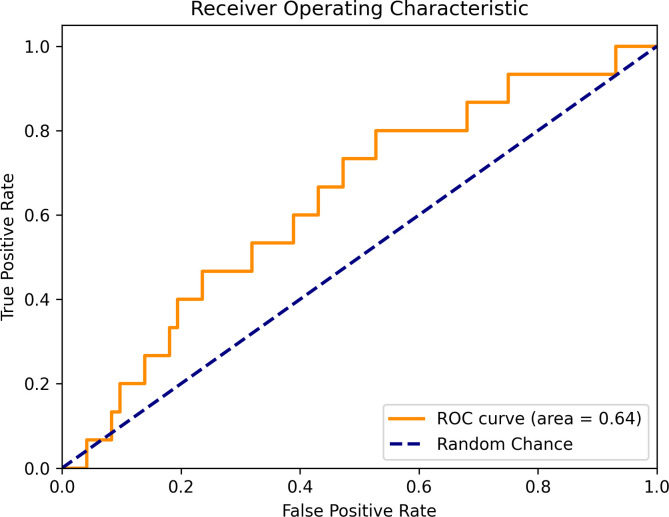
Down sampled XGBoost ROC curve.

The down sampled XGBoost model performed the best on the test data by having the highest F-1 score and AUC score compared to all other machine learning models, so it was further explored Fig 2. represents the ROC curve of the down sampled XGBoost model. Overall, the ROC curve lies above the random prediction dotted line, except for a small segment that dips below the random prediction line.

This indicates that at a few thresholds, the model’s true positive rate was lower than expected compared to the false positive rate. Despite this, most of the ROC Curve is above random prediction and with an AUC score of 0.64, the down sampled XGBoost model performs better than random chance.

**Fig 3:**
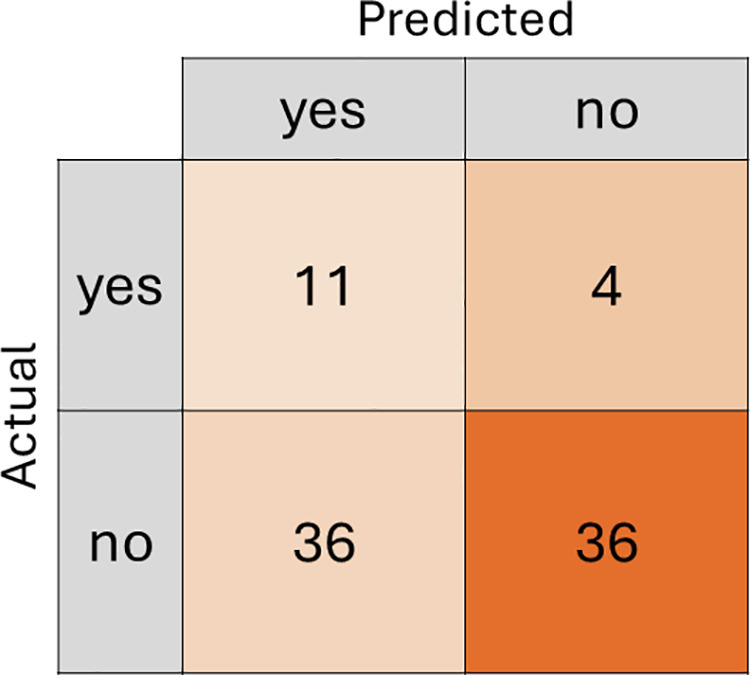
Down sampled XGBoost confusion matrix prediction on test data.

The confusion matrix (Fig 3) shows that the model correctly identified 11 people who left the program (true positives) and 36 people who stayed (true negatives). However, the model predicted that 4 people would stay in the program when they actually left (false negatives) and predicted 36 people to leave when they stayed (false positives). While this dataset was balanced, the higher misclassification of the positive class may indicate residual bias.

**Fig 4:**
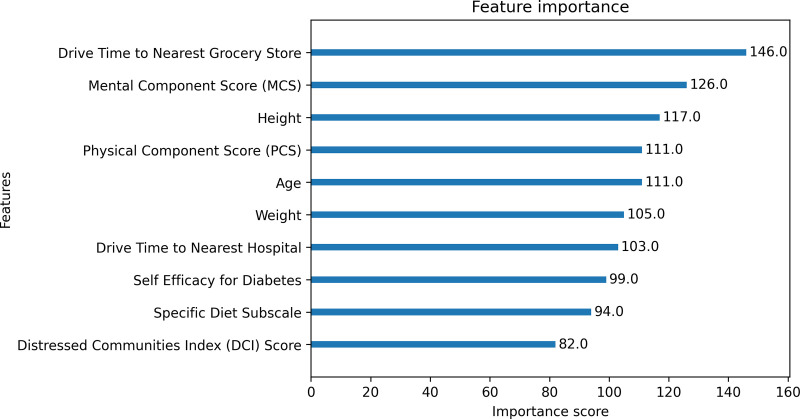
Top 10 features from down sampled XGBoost Model.

Fig 4 illustrates the top 10 variables with the highest gain, or contribution of a feature improving the prediction of attrition. A higher gain indicates features that reduce the loss of a prediction when that feature is used to split a decision tree.

There are a few immediate themes with the features ranked as the most influential. Mental Component Score (MCS) and Physical Component Score (PCS) both were ranked highly from the SF-12 data. Demographic variables such as age, weight, and height were also deemed influential. Lastly, calculated geographic variables (drive time to nearest grocery store and drive time to nearest hospital), diabetes management characteristics (self-efficacy for diabetes and specific diet subscale) and DCI score were featured.

**Fig 5:**
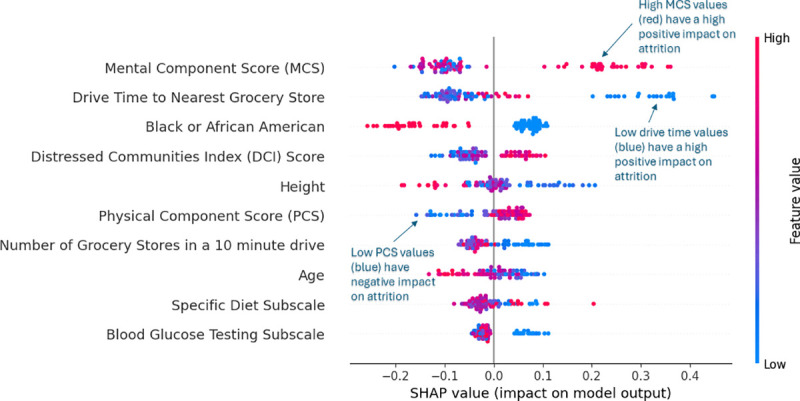
SHAP value beeswarm plot for top 10 features.

While ranking feature importance using gain is helpful in highlighting influential features, it is difficult to evaluate how those features impacted attrition. To assist with model interpretation, Fig 5 represents a SHAP beeswarm plot of the top 10 most impactful features by average SHAP value on predicting attrition on the test data. The higher the feature in the plot, the more impactful they were. In the SHAP beeswarm plot, red points indicate instances with higher values, while blue values indicate instances with lower values of the feature.

Compared to Fig 4, Fig 5 shows a slightly different variable importance ranking, which can be more representative of the features’ contribution to the model prediction because SHAP calculates the marginal contribution of each feature to each prediction. Interestingly, MCS, drive time to the nearest grocery store, whether someone was black or not, and DCI score were shown to be the top four most important features, respectively. MCS was the most important feature, where higher MCS values were associated with a higher likelihood of dropping out of the diabetes program.

**Fig 6:**
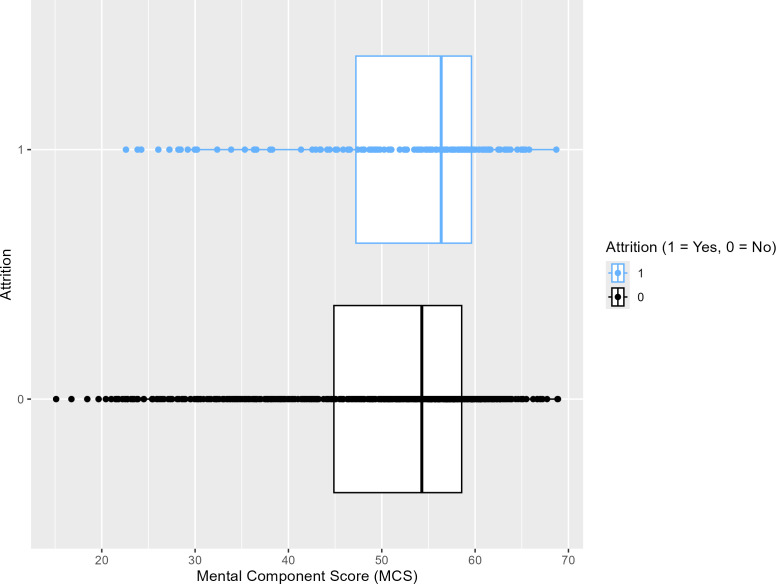
Box plot comparing people’s mental component scores by attrition status.

Fig 6 displays a box plot illustrating that participants who dropped out of the diabetes program (blue) had slightly higher MCS scores compared to those who remained in the study (black). This visual pattern is consistent with our SHAP values findings, which identified MCS as an important predictor of attrition. A Mann-Whitney U test revealed a statistically significant difference in MCS scores between the two groups (*P* = 0.03), indicating that higher MCS values were associated with program dropout.

**Fig 7:**
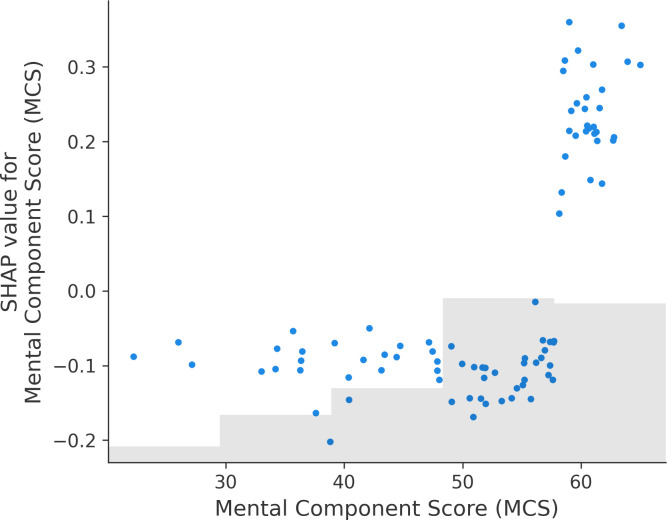
Scatter plot of mental component score and SHAP value per instance.

In addition, Fig 7 presents a SHAP dependence plot illustrating the relationship between MCS scores and their corresponding SHAP values across all instances. SHAP values remain relatively flat for MCS scores below 60, indicating a low predicted risk of attrition. However, for participants with MCS scores above 60, SHAP values increase sharply, suggesting a higher likelihood of leaving the program.

A minor pattern was also observed indicating that participants with shorter drive times to grocery stores were more likely to drop out. Furthermore, race emerged as an influential factor, with Black or African American participants (1 = Black, 0 = not Black) being less likely to leave the program. Lastly, higher DCI scores were associated with increased attrition risk. These findings underscore the combined impact of individual health metrics, environmental proximity, and demographic characteristics in predicting program attrition, highlighting the multifaceted nature of participant engagement.

## Discussion

Current application of machine learning in diabetes research primarily focuses on early detection and risk prediction, with a paucity of literature utilizing machine learning to predict program engagement and attrition. The goal of this study was to assess the predictive effectiveness of several machine learning models for attrition from a diabetes self-management program using participant demographics and various evaluation measures. Through these models, several variables emerged as important predictors of attrition, including health-related measures (quality of life scores, DCI score, specific diet and blood glucose testing subscales), demographic factors (race, age, weight, height, and educational attainment), and spatial variables (drive time to the nearest grocery store and the number of grocery stores within a ten-minute radius), suggesting that both individual- and community-level factors may play a role in program attrition. All models had low AUC (0.53 to 0.64) and F-1 scores (0.19 to 0.36), indicating poor predictive performance. However, Mann-Whitney U and odds ratio revealed significant associations with attrition, suggesting possible insights into contributing factors. Imbalanced data, with more participants completing the program than dropping out, posed a challenge, making the models prone to overfitting. Applying balancing techniques slightly improved model performance. Notably, down sampled XGBoost performed the best, indicating the highest ability to distinguish between individuals’ program attrition status. These findings highlight the complexity of predicting behavior-based outcomes, and suggest that, despite their limited predictive accuracy, machine learning may still be able to aid in identifying factors relevant to reducing program attrition.

The logistic regression conducted in this study found that race and education were observed to be significantly associated with attrition from a diabetes self-management program. White participants were significantly more at risk of dropping out of HED compared to African American participants. This finding differs from current literature, which indicates that African American and other minority populations have lower retention rates compared to White participants [43]. Health Extension for Diabetes is a community-based diabetes self-management program that focuses on creating culturally tailored programs toward its participants. Additionally, HED facilitators who lead the program are typically members of the community and understand their participants’ beliefs and barriers. Previous literature has observed that culturally tailored diabetes management programs improve program participation [44,45]. The results from this study may support these findings and could indicate that diabetes self-management programs that are culturally tailored may result in better retention rates for minority populations compared to a standard care program.

Similar to the logistic regression model, the XGBoost indicated that individuals who were less vulnerable were more likely to drop out of HED. The XGBoost model identified MCS scores as the variable with the most significance in predicting attrition. A trend emerged suggesting that people with better mental and physical health were more likely to drop-out of the program. Previous literature reported that individuals in HED with low and low-to-moderate social vulnerability scores, as measured by the Social Vulnerability Index, did not show significant improvement in their mental health pre- to post-HED, while individuals with moderate-to-high and high social vulnerability scores showed significant improvements in their mental health [46]. Additionally, the XGBoost identified that individuals who were White, had a lower drive time to the nearest grocery store and lived in a less distressed zip code were more likely to drop out of HED. Taken together, these findings suggest that diabetes self-management programs designed for vulnerable populations may be less effective in engaging individuals with lower social vulnerability, potentially due to differences in perceived benefit, relevance, or unmet expectations. This underscores the need for future research to examine how program content and delivery can be adapted to retain a broader spectrum of participants.

There is a paucity of literature using machine learning to predict attrition in diabetes self-management programs. While this study attempted to address this gap, the machine learning models explored demonstrated poor predictive performance, with AUC values ranging from 0.53 to 0.63. In healthcare, datasets are commonly imbalanced due to the rarity of more complex conditions [47]. After exploring multiple balancing techniques on this dataset, the poor predictive performance could suggest a weak correlation between the program participants’ characteristics and attrition rather than data imbalance alone. These findings could indicate that the commonly collected demographic and pre-post program evaluation measures may not be sufficient for identifying individuals at-risk of dropping out of diabetes self-management programs. This insight could inform future data collection and program evaluation efforts, emphasizing the need to integrate more nuanced factors. Further research is necessary to explore additional features that could influence attrition risk and conduct additional studies with larger sample sizes.

## Limitations

To improve model performance, additional data is needed. A key limitation of this analysis is the dataset, with only 16% of participants dropping out, which may affect model performance. Furthermore, some critical factors influencing program attrition could be missing, limiting the comprehensiveness of the analysis. Another limitation is the generalizability of the findings. The sample population is from South Carolina, a state with one of the highest diabetes rates in the United States. The influences affecting this population may differ from those in other geographical regions, impacting the applicability of the results elsewhere. However, HED was designed for nationwide implementation, adapting to the cultural, geographic, and resource differences of diverse communities. Ongoing research is evaluating HED’s effectiveness across various states, and initial findings suggest successful program outcomes beyond South Carolina, helping to minimize this limitation.

## Conclusions

This study aimed to evaluate the effectiveness of machine learning models for predicting attrition from a diabetes self-management program, based on participant demographics and evaluation measures. Despite exploring multiple machine learning models, all exhibited poor model performance (AUC values between 0.53 and 0.64). While these models were not suitable for accurately predicting individual attrition risk, they highlighted potential factors influencing dropout rates. The XGBoost when balanced using the down sampling technique, showed the best performance among the models tested.

Through the various methods utilized in this study, health-related measures (quality of life scores, DCI score, specific diet subscale, and blood glucose testing subscale), demographic factors (race, age, weight, height and educational attainment), and spatial variables (drive time to nearest grocery store and number of grocery stores within a 10-minute drive) emerged as significant predictors of attrition. While the models achieved only modest predictive power, a trend was observed across the important features that indicated that those in less vulnerable populations were more likely to drop out of HED. This finding may indicate diabetes self-management programs tailored to vulnerable populations may not be addressing the needs of those in populations with lower risk. Future research should explore how program design can be adapted to better engage lower-risk individuals while continuing to support vulnerable populations.

The prevalence of imbalanced data in healthcare modeling remains a challenge, limiting the practical implementation of machine learning in clinical settings. In this study, balancing the machine learning models only marginally improved prediction, suggesting that the data imbalance alone may not fully explain poor model performance. Previous literature has largely focused on using machine learning to predict diabetes onset based on various risk factors, with limited research applying these methods to predict attrition from diabetes management programs. These findings underscore the difficulty of predicting health behavior outcomes and highlight the need for future research to improve model performance by incorporating additional behavioral and contextual predictors and expanding sample sizes to better support patient engagement and retention.
